# Concomitant pulmonary and thyroid tumors identified by FDG PET/CT and immunohistochemical techniques

**DOI:** 10.1186/1477-7819-9-119

**Published:** 2011-10-06

**Authors:** Guangwen Zhu, Hong Li, Yanjun Zhang, Yaming Li, Shujun Liang, Jia Liu

**Affiliations:** 1 Department of Nuclear Medicine, the First Affiliated Hospital, Dalian Medical University, Dalian, China; 2 Liaoning Laboratory of Cancer Genomics and Department of Cell Biology, Dalian Medical University, Dalian, China; 3 Department of Nuclear Medicine, the First Affiliated Hospital, China Medical University, Shenyang, China; 4 Department of Nuclear Medicine, the Second Workers' Hospital of Liaohe Oilfield, Panjin, China

## Abstract

**Background:**

The exact diagnosis of double primary papillary adenocarcinoma of thyroid and lung is even rarer, to our knowledge no report in the literature by [^18^F]-2-fluoro-2-deoxy-D-glucose-positron emission tomography/X-ray CT(FDG PET/CT) with surgical specimens immunohistochemistry(IHC). We report a patient with abnormal FDG PET/CT in thyroid and lung, this unusual presentation may lead to misdiagnosis without surgical specimens IHC.

**Case presentation:**

A 56-year-old man with coughing three months. FDG PET/CT was performed, and resection specimens of lung and thyroid were detected by hematoxylin eosin staining (HE) and IHC. PET/CT: lung tumor SUVmax: 3.69, delay: 5.17; and thyroid tumor SUVmax 19.97. HE reveal papillary adenocarcinoma, but histological differentiation of primary pulmonary adenocarcinoma from metastatic adenocarcinoma is sometimes difficult because of their phenotypic similarities. So IHC was performed, the IHC of lung tumor: cytokeratin 20 (CK20)(-), thyroglobulin(Tg)(-), cytokeratin7(CK7)(+), thyroid transcription factor-1 (TTF-1)(+); thyroid tumor: CK7(+), TTF-1(+), thyroglobulin (+), CK20(-). Therefore, the final diagnosis was double primary adenocarcinomas of thyroid and lung.

**Conclusion:**

FDG PET/CT has preliminary diagnostic capacity of multiple primary tumors; the final diagnosis should be adopted for specimens after tumor-specific markers IHC to obtain. Consequently, effective therapeutic approaches can be designed and conducted.

## Background

Early detection and correct diagnosis are essential for definite treatment and better outcome of cancer patients. FDG PET/CT scans can detect thyroid incidentalomas of which 33.2%-63.6% are found with malignant phenotypes, and a body of evidences has shown the effectiveness of PET/CT in differential diagnosis of benign and malignant tumors [1-3]. Nevertheless, it remains difficult to distinguish multiple primary tumors from the metastatic ones with this approach and, therefore, fine-needle aspiration (FNA) and cytological examination have to be employed in the final diagnosis. Malhotra G et al [4] found bronchoalveolar carcinoma of lung masquerading as iodine avid metastasis in a patient with minimally invasive follicular thyroid cancer. However, their conclusion just only based on the IHC of CT-guided biopsy, and the diagnostic accuracy of core biopsy is lower than surgical excision specimen. Eloy JA et al [1] reported that incidental FDG uptake in the thyroid gland of the patients with nonthyroidal cancers was associated with a 27.8% risk for well-differentiated thyroid carcinoma, but they did not provide technical detail about differential diagnosis of multiple primary tumours or metastatic diseases. The current tumor classification is largely based on HE histological staining, but the lining cells in pulmonary papillary adenocarcinomas show cuboidal to columnar phenotypes, an outlook similar to the papillary carcinoma of the thyroid. In such case, IHC, in addition to conventional histopathological examination, would be required to distinguish primary and metastatic adenocarcinomas from the double primary cancers derived from different cells but in similar phenotypes.

## Case presentation

**Figure 1:**
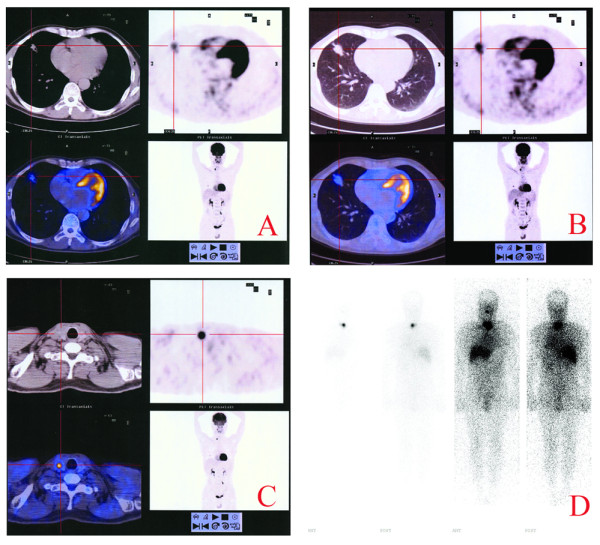
**Whole-body FDG PET/CT and radioiodine scan. **A hypermetabolic focus in the mid lobe of lower lobe of the right lung with increased FDG uptake in SUV max 3.69 (A) and delayed SUV 5.17 (B). PET/CT-detected round hypermetabolic focus in the right lobe of the thyroid with abnormal FDG uptake in SUVmax 19.97(C). Postoperative therapeutic dose ^131^I whole body imaging showed mild accumulation of radioiodine (T/NT 3.64) in the residuary thyroid, while no abnormal ^131^I uptake in the other side (D).

**Figure 2:**
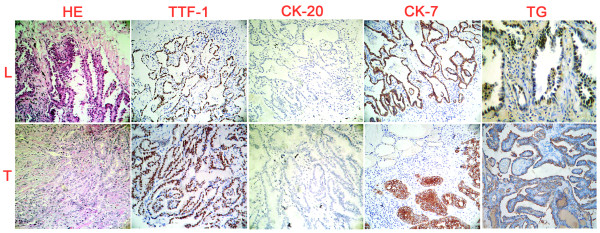
**HE histological staining of the resected lung (L) and thyroid tumor tissue (T): The lining cells in resected lung tumor tissue were cuboidal to columnar, similar to the papillary carcinoma of the thyroid**. IHC staining: negative immunoreactivity of CK20 and thyroglobulin but positive of CK7 and TTF-1 in the lung tumor; positive immunoreactivities of CK7, TTF-1 and thyroglobulin but not CK20 were found in the thyroid tumor.

We report a 56-year-old man who had suffered from cough, chest tightness and shortness of breath for 3 months and became more aggravated for recent 3 weeks, without hoarseness or loss of voice, haemoptysis, weight loss, bone pain, abdominal pain, the history of thyroid disease and neurological symptoms. X-ray CT examination detected a nodule in the right mid lobe of the lung in the maximal diameter of 2.21 cm and irregular density, complementing with pleural retraction. The CT value of this nodule was 42HU, the contrast enhancement CT value was 70HU and the delayed one was 81HU. Since the location of this nodule was beyond the reach of fiberbronchoscopy forceps for cytological examination and clinical staging, FDG PET/CT (GE Discovery ST, USA) was performed, which showed the increased FDG uptake of the tumor in terms of the SUVmax in 3.69 and the delay SUV in 5.17 (Figure 1A and 1B), and no abnormal FDG uptake was found in the nodule-free lung tissues and mediastinum space. It was also found that in the right lobe of the thyroid, there was a round hypermetabolic focus in 1 cm maximal flow path diameter, which showed FDG uptake of SUVmax in 19.97, low CT density and ambiguity of the border (Figure 1C). This patient was therefore hospitalized and subjected to a right lung mid lobe lobectomy and mediastinal lymph node dissection. According to the pathological examination, the surgical specimen was elastic-firm and composed of the tumor cells that arranged in papillary configuration or fused glandular structures with irregularly enlarged hyperchromatic nuclei (Figure 2). All of the six dissected mediastinal lymph nodes were free of tumor cells. This specimen was thus diagnosed as a well-differentiated pulmonary adenocarcinoma without lymph node metastasis.

After the operation, the patient was treated by conventional adjuvant chemotherapy with paclitaxel, cisplatin and vinorelbine tartrate for 4 cycles/courses. Six months later, this patient was re-admitted for right lobe thyroidectomy. The microscopic examination revealed a papillary structure of the removed thyroid tissues with a ground-glass appearance of tumor cell nuclei, irregular nuclear contours and some colloid within neoplastic follicles (Figure 2). Based on these pathological findings, the removed specimen was diagnosed as papillary carcinoma of the thyroid.

Sensitive and reliable markers such as TTF-1, Tg, CK7 and CK20 were used to further ascertain the origin(s) of the two tumors co-existing in the lung and the thyroid. TTF-1 and Tg are considered as markers for differential diagnosis in distinguishing primary tumor of the thyroid or lung from other origins. CK7 and CK20 are high molecular weight cytokeratins, the different expression patterns of CKs allow the accurate and sophisticated classification of epithelial cells and their neoplasms into different subtypes. So the IHC staining for TTF-1, CK20, CK7 and Tg were performed on the two tumor specimens(Figure 2), which revealed negative immunoreactivity of CK20, Tg but positive of CK7, TTF-1 in the tumor removed from the lung; while positive immunoreactivity of CK7, TTF-1, Tg but negative of CK20 in the tumor removed from the thyroid. These results suggested that this patient bear double primary adenocarcinomas originated from the lung and the thyroid, respectively. Three months after the second operation, a therapeutic dose of ^131^I (100mCi) was adopted for ablation thyroid remnant; 7 days later, the scintigraphy showed mild accumulation of radioiodine (target/nontarget ratio: T/NT 3.64) in the residuary thyroid, while no abnormal ^131^I uptake was found (Figure 1D). The patient was followed up for 3 years. Comprehensive examination revealed no sign of recurrence and metastasis and the general physical state of the patient is well kept.

## Discussion

The diagnostic sensitivity and specificity of FDG PET/CT scan has been fully validated in cancers. But with a multiple abnormal FDG PET/CT scan, it is hard to make a definite differential diagnosis of primary or metastatic ones, and CT also can not distinguish primary tumors from metastatic ones, for example, solitary pulmonary metastases similar to primary lung tumor. So a multiple abnormal FDG PET/CT should lead to reevaluation of the initial diagnosis if the patient with a high risk for secondary neoplasia. In this case, FDG PET/CT scan detects two nodules in lung and the thyroid, respectively, but can not make a final diagnosis about concomitant pulmonary and thyroid tumors or metastatic ones. Therefore, the following possibilities could be taken into account and should be clarified by appropriate approaches.

The possibility of lung adenocarcinomas (L-ACs) with thyroid metastases was considered firstly. The L-ACs are one of the most frequent malignancies, which have strong tendency of distant metastasis via lymphatic or hematogenous routes. The thyroid gland is a common target organ of metastasis because of its rich vascularization structure, and majority of the secondary thyroid tumors are originated from the carcinomas of the breast (8.8%), the stomach (7.7%) and especially the lungs (43%) [5]. Histological differentiation of primary thyroid cancers from metastatic L-ACs is sometimes difficult because of their phenotypic similarities. L-ACs usually exhibit cytoplasmic immunoreactivity of CK7 and negative CK20, while primary thyroid carcinomas show thyroglobulin production [6]. In this case, the diagnosis of L-ACs with thyroid metastasis can not be established because of the positive immunolabeling of CK7, TTF-1 and Tg in the thyroid specimen.

Another possible diagnosis of this case was the thyroid adenocarcinoma(T-AC)with lung metastases. As a malignant epithelial tumor in the synonym of papillary adenocarcinoma, T-ACs usually spread to regional lymph nodes and, sometimes, form metastatic foci and nodules in other organs including the lungs [7]. The lung metastases from thyroid cancer are positive in thyroglobulin, while this protein is negative either in primary lung cancers or in the tumors originated from other sites [6]. In this case, since the tumor specimen removed from the lung was negative in CK20 and Tg but positive in CK7 and TTF-1, it can be diagnosed as primary adenocarcinoma of the lung rather than lung metastasis from thyroid cancer.

Synchronous metastases to the lungs and the thyroid can be found in clinic. FDG PET/CT can provide valuable information to establish this diagnosis by showing other tumor(s) in addition to the nodules in the thyroid and lungs. In this case, since no tumor was detected besides lung and thyroid nodules via FDG PET/CT scan, the possibility of synchronous metastases to the lungs and the thyroid could be ruled out.

If with synchronous metastases, it is usually difficult to determine the tissue origin of metastatic adenocarcinoma simply based on their histopathological features. In such case, IHC using specific biomarkers for individual cancers would be informative in identifying tumor origins. For example, TTF-1 and surfactant proteins (SP-A, pro-SP-B, pro-SP-C) can be used to identify lung cancer, thyroglobulin to thyroid, prostate specific antigen to prostate, mammaglobin 1 to breast, pepsinogen C to stomach, metallothionein IL to pancreas, uroplakin II to bladder, MUC II to colon cancers [8].

Concomitant pulmonary and thyroid primary adenocarcinomas or multiple raised from different organs are also possible such as the double adenocarcinomas of the lungs and thyroid [9]. The incidences of multiple primary malignancies were about 11% and 7% among the patients with overall and resected non-small cell lung carcinomas [10]. In this case, based on the findings of FDG PET/CT and IHC staining, double primary cancers are highly speculated and finally determined, and more definite remedies were thus designed for the primary lung and thyroid adenocarcinomas of the patient, which achieved desirable therapeutic results in terms of cancer-free status for 3 years.

To our knowledge, this is the first report of a patient who was diagnosed with concomitant pulmonary and thyroid primary adenocarcinomas by FDG PET/CT and IHC approaches. Using ''double primary papillary adenocarcinoma of lung and thyroid'' as keywords, the PubMed Database yielded one similar case, but the diagnosis of that report didn't based on IHC approaches, only by HE.

## Conclusion

FDG PET/CT has preliminary diagnostic capacity of multiple primary tumors. The final diagnosis, only with the surgical specimens, can be made based on the combination of histological evaluation and IHC for the biomarkers specific to individual tissue or cancer types. Consequently, effective therapeutic approaches can be designed and conducted.

## Consent

Written informed consent was obtained from the patient for publication of this case report and accompanying images. A copy of the written consent is available for review by the Editor-in-Chief of this journal.

## Competing interests

The authors declare that they have no competing interests.

## Authors' contributions

GZ: guarantor of integrity of the entire study; JL: study concepts and design; HL: experimental studies/data analysis; YZ: manuscript preparation; YL: literature research; SL: manuscript editing. All authors read and approved the final manuscript.
